# Water quality and immatures of the M and S forms of *Anopheles gambiae *s.s. and *An. arabiensis *in a Malian village

**DOI:** 10.1186/1475-2875-5-35

**Published:** 2006-04-29

**Authors:** Frances E Edillo, Frederic Tripét, Yeya T Touré, Gregory C Lanzaro, Guimogo Dolo, Charles E Taylor

**Affiliations:** 1 Department of Ecology and Evolutionary Biology, University of California at Los Angeles, CA 90095-1606, USA; 2 Harvard School of Public Health, Department of Immunology and Infectious Diseases, Boston, MA 02115, USA; 3 Vector Genetics Laboratory, Department of Entomology and Center for Vectorborne Diseases, University of California at Davis, Davis, CA 95616, USA; 4 Center for Applied Entomology and Parasitology, School of Life Sciences, Keele University, Staffordshire, ST5 5BG, UK; 5 Malaria Research and Training Center, Département d'Epidémiologie des Affections Parasitaires, Faculté de Médecine de Pharmacie et d'Odonto-Stomatologie, Bamako, B.P. 1805, Mali; 6 Special Program for Research and Training in Tropical Diseases (TDR), WHO, 1211 Geneva 27, Switzerland

## Abstract

**Introduction:**

The associations between the immatures of *Anopheles gambiae *s.s. (Diptera: Culicidae), its M and S forms, and *Anopheles arabiensis *among and within larval breeding habitats in Banambani, Mali were investigated under varying conditions of water quality and rainfall. The intent was to elucidate on niche partitioning of these taxa.

**Methods:**

Immatures of *An. arabiensis, An. gambiae *s.s., and its M and S forms were sampled every alternate day for a month in mid-rainy season from three sampling sites in each of the larval breeding habitats (rock pools, swamp, and puddles). Water quality was characterized by alkalinity, conductivity, dissolved oxygen (D.O.), nitrate, orthophosphate, pH, temperature, total dissolved solids (TDS), and turbidity. A type 3 analysis of the GENMOD model was used to examine the associations between the proportional frequencies of young (first and second instar larvae) and old (third and fourth instar larvae and pupae) or total immatures of species or forms among sampling sites within and among larval breeding habitats during a category of rainfall as influenced by water quality.

**Results:**

Of the 4,174 immatures sampled, 1,300 were molecularly identified to species and forms. Significant association between the proportional frequencies of young larvae of *An. arabiensis, An. gambiae *s.s., its M and S forms was found among sampling sites within habitats but not among larval breeding habitats. The proportional frequencies of young larvae of M and S forms varied daily perhaps due to recruitment, mortality, and dispersal within habitats. Conductivity and TDS had significant effects when the proportional frequencies of young larvae of M and S forms among sampling sites within habitats were significantly associated. Alkalinity, D.O., orthophosphate, pH, nitrate, temperature and turbidity had no effects on niche partitioning of species and forms among sampling sites within habitats. Rainfall did not affect the frequencies of these immatures.

**Conclusion:**

Conductivity and TDS have significant effects on niche partitioning of young larvae of M and S forms among sampling sites within habitats in Banambani, Mali.

## Background

The genetic structure of the *Anopheles gambiae *s.l. complex is thought to have enabled members of this complex species to occupy different habitats [1-5]. The two principal vectors of malaria in this group, *An. gambiae *s.s. and *Anopheles. arabiensis*, are broadly sympatric but there are areas where only one or the other may be found [6]. Macrogeographic studies of habitat usage across sub-Saharan Africa generally find that *An. gambiae *s.s. and *An. arabiensis *differ principally along axes of warm-cool and dry-wet, though there is more to differences than just climate [7-11]. Less is known about macrogeographic differences across sub-Saharan Africa in habitat usage among chromosomal or molecular forms of *An. gambiae *s.s. though Touré *et al*. [12] reported that they exist. The S form is distributed from 13°W to 50°E and is the only form recorded mainly in the east of Great Rift Valley, whereas the M form seemingly shows a greater distribution from 16°N to 16°S and is the only form found in savannah areas of northern Senegal and in the desert borders of southern Angola [13]. In west Africa, the M form seems able to exploit man-made habitats that are otherwise available only to *An. arabiensis *in dry areas [5].

Much less is known about microgeographic differences in habitat usage by *An. arabiensis, An. gambiae *s.s., and its M and S forms. The location of larvae in a habitat is due to selection of oviposition site by gravid females, and numbers available for sampling depend upon larval dispersal and survival. In Mali, *An. gambiae *s.s. and *An. arabiensis *often coexist together, and each occupies a variety of habitats. Charlwood and Edoh [10] observed differences between the larval distribution of *An. gambiae *s.s. and *An. arabiensis *based on distance from adult feeding sources in Tanzania. Minakawa *et al*. [11] observed that the distance to the nearest house and types of soil substrate were significantly associated with the relative larval abundance of *An. gambiae *in Kenya, but were unable to detect any significant association between their occurrence and larval habitat variables. Gimnig *et al*. [14] reported that *An. gambiae *s.l. was associated with turbid water, algae, the absence of emergent vegetation, and small habitats. In laboratory studies, McCrae [15] observed that *An. gambiae *s.l. laid more eggs in petri dishes with turbid water from a natural site than in distilled or tap water. Gimnig *et al*. [16] found that adequate food such as algae, bacterial composition and nitrogen were important regulators of larval growth of *An. gambiae*. These studies were directed at understanding species-level differences in habitat usage within the *An. gambiae *complex, and did not attempt to distinguish the habitat usage of the chromosomal or molecular forms within *An. gambiae *s.s.

In an earlier study [17], habitat differences between species (*An. arabiensis *vs. *An. gambiae *s.s.) and between molecular forms (M vs. S) of *An. gambiae *s.s. were explored in Banambani, Mali. Differences were greater between species than between the M and S forms among the larval breeding habitats (rock pools, the swamp, and puddles). The larval abundance of *An. gambiae *s.s. and *An. arabiensis *differed significantly among habitats. Differences between the immatures of the M and S forms were found among habitats in Banambani, Mali in 1999 when rainfall was high, but not during the two drier sampling periods in 1997–1998. In Burkina Faso, the M form larvae occurred at a higher relative frequency than the S form in man-made or more permanent habitats (e.g. irrigated areas), whereas the S form larvae were more prevalent than the M form in more natural sites (e.g. rain-dependent pools) [5,13,18,19]. Spatial segregation between the M and S forms was not influenced by their differences in exploiting the physicochemical conditions of their larval breeding sites [20]. Ayala and Coluzzi [21] predict that there must be specialization of the different forms to separate niches prior to complete reproductive isolation.

In the current study, the strength of associations between the proportional frequencies of the immatures of *An. arabiensis *and *An. gambiae *s.s., or its M and S forms among the larval breeding habitats (rock pools, the swamp, and puddles), or among sampling sites within a habitat in Banambani, Mali was examined under varying conditions of water quality, and rainfall during a mid-rainy season. The strength of associations between the young (first and second instar larvae) and old (third and fourth instar larvae and pupae) or total immatures of either species or forms within and among habitats was also determined as influenced by each of the physicochemical variables of water. This was motivated, in part, by differential mortality among life stages of *An. gambiae *s.l. in different aquatic habitats [22]. The intent was to shed further light on ecological niche partitioning that had been observed earlier.

## Materials and methods

### Larval breeding habitats and sampling sites within habitats

**Figure 1:**
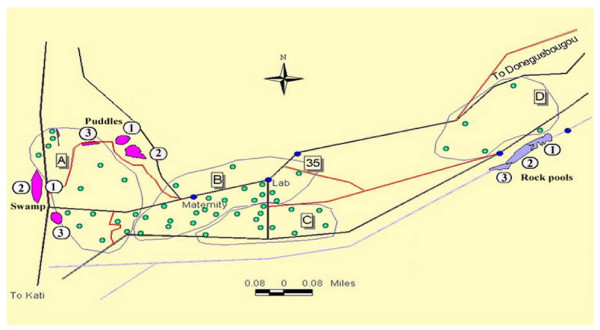
An updated map of Banambani village, Mali during the rainy season of 2000. Encircled numbers are the three sampling sites in each of the larval breeding habitats (rock pools, swamp, and puddles). Shaded circles are the compounds where villagers live in huts.

Banambani village, the study site, is about 20 km northeast of Bamako, in the Northern Sudan Savanna of Mali, West Africa (12° 48' N and 8° 03' W). For a more detailed description and pictures, see Edillo *et al*. [17,23]; Touré *et al*. [12,24]; Touré [25]. An updated map of Banambani village, indicating locations of the larval breeding habitats and sampling sites within each habitat, is shown in Figure 1. Larval breeding habitats around Banambani have been monitored since 1997 [17,23]. For this study, three sampling sites were established within each habitat for regular collection of immatures and monitoring of water quality. The fieldwork was conducted during the rainy season, 28 July – 25 August, 2000. The weather data during this period were: 237.2 mm of total rainfall in 15.8 h and 27.3°C ± 0.65 mean monthly temperature ± S.E.

### Sampling of immatures

Larvae and pupae of *An. gambiae *s.l. were sampled every alternate day from 28 July to 25 August, 2000 whenever weather permitted. A sampling consisted of 60 dips (350 ml) made using a standard aquatic larval dipper from near the edges of each sampling site within each habitat. Immatures were stored in plastic bags containing water from the sampling site and brought to the laboratory in Bamako on the same day they were collected, where they were individually placed dry into 1.5-ml microcentrifuge tubes and were stored at -80°C until processed for DNA extraction.

### PCR

*An. gambiae *s.s. was distinguished from *An. arabiensis *by the polymerase chain reaction-based diagnostic of Scott *et al*. [26]. Larvae and pupae samples were randomly chosen from each sampling site within each habitat on each sampling day. DNA was extracted following a procedure modified from that of Lanzaro *et al*. [27] and described in Edillo *et al*. [17]. DNA templates were amplified with primers specific for each sibling species as described by Scott *et al*. [26]. Aliquots of randomly selected individuals identified as *An. gambiae *s.s. were used to separate M from S molecular forms following the PCR-based diagnostic of Favia *et al*. [28].

### Water quality within each habitat

The physicochemical properties of water samples from which immatures of *An. gambiae *s.l. were sampled from the edge of each sampling site within each habitat were measured between 0900–1200 h with a LaMotte water chemistry kit (LaMotte Company, Chestertown, MD) following procedures described in The Monitor's Handbook [29]. Measurements of conductivity, dissolved oxygen (D.O.), nitrate (NO_3_), orthophosphate, pH, total alkalinity, total dissolved solids (TDS), turbidity, and water surface temperature are described in Edillo *et al*. [23]. Concentrations of D.O. (ppm), nitrate (ppm), orthophosphate (ppm), and total alkalinity (ppm) were measured weekly; turbidity expressed in Joules turbidity unit (JTU) was monitored biweekly; conductivity (μmhos/cm) and TDS (ppm) were determined every other sampling day during the second and fourth week of the fieldwork. Water temperatures (°C) and pH at the edge of each sampling site were determined at each sampling day. The mean ± S.E. of all repeated measurements of each physicochemical property of water in each larval breeding habitat or sampling sites within each habitat were calculated.

### Rainfall

Prior studies had indicated that rainfall seems to be a critical feature of habitat differences, especially between the molecular forms of *An. gambiae *s.s. [17]. Heavy rainfalls over a short period may create new pools and flush larvae out of some sites, moderate rains distributed over the wet season probably prolong the life of temporary pools and facilitate more eggs and larvae to mature [30]. Thus, the count data of mosquito immatures were divided into three categories of rainfall [23]: 1) samples collected one day after a heavy rainfall, 2) after a moderate rainfall, and 3) no rain for at least two days.

### Data analysis

The count data for species (*An. gambiae *s.s. vs. *An. arabiensis*) and forms of *An. gambiae *s.s. (M vs. S) were divided into three groups: 1) young larvae made up of first and second instar larvae, 2) old immatures composed of third and fourth instar larvae and pupae, and 3) total immatures. The strength of association between the species or forms (the response) and larval breeding habitats or sampling sites within a habitat in each day's sampling and during a category of rainfall (the predictors) was examined by repeated measures logit model using a type 3 analysis of the GENMOD procedure [31,32]. This test computes the likelihood ratio statistics for the overall main effects of predictors on the proportional frequencies of these groups of immatures, and ensures that results do not depend on the order specified in the model. The model does not treat the absence or presence of either species or forms. However, it calculates the ratio of *events/trials*, where *event *is the response of interest (e.g., number of young vs. old immatures of *An. gambiae *s.s. and *An. arabiensis*, or of the M and S forms), and *trials *is the total number of possible events (e.g., total number of young and old immatures of species, or of forms). Because the data are nested (i.e., in each of the 14 collection days, there are data from three sites in each of the three habitats), covariance-variance structure in the *repeated *statement of the model is incorporated to correct for potential problem of correlated data points.

The strength of association between each physicochemical property of water, to avoid multicollinearity, and the proportional frequencies of *An. gambiae *s.s. and *An. arabiensis*, or the M and S forms (young, old and total immatures) among larval breeding habitats and sampling sites within a habitat, and during a category of rainfall was further examined by using the above model. For analyses that showed insignificant effect of a physicochemical property of water but had significant association between the proportional frequencies of species or forms among sampling sites within habitats but not among larval breeding habitats, the GENMOD model was modified by eliminating the habitat effect.

## Results

### Physicochemical characteristics of water in larval breeding habitats and sampling sites within each habitat

The mean physicochemical characteristics of water among the sampling sites within each habitat are shown in Table 1. Turbidity varied significantly among sampling sites within each habitat (*F *= 12.82, *df *= 2, *P *< 0.01) but not among larval breeding habitats (*F *= 1.71, *df *= 2, *P *> 0.05). Water was relatively turbid (265 ± 65 JTU) among sampling sites in the swamp, and clear or slightly turbid (30 ± 5 JTU) in rock pools. The three puddle sites varied in their turbidity from clear (20 ± 5 JTU) to turbid (105 ± 5 JTU) or very turbid (330 ± 30 JTU). Conductivity and TDS varied among the three puddle sites and among two sampling sites in the swamp and rock pools. Immatures were collected in water with pH values ranging from slightly acidic to basic (Table 1). All puddles, rock pools, and sampling site 3 of the swamp were exposed to the sun, whereas sampling sites 1 and 2 of the swamp were partly shaded by surrounding trees.

**Table 1:** Mean (± S.E.) and range (enclosed in parentheses) of physicochemical characteristics of water samples in sampling sites within a habitat in Banambani

**Habitat - **Site	Alkalinity (ppm)	Conductivity (μmhos/cm)	D.O. (ppm)	Ortho-phosphate (ppm)	pH
Rockpools					
1	23.75 ±1.9 (18 – 38)	55.7 ± 19.7 (36 – 75.4)	8.34± 0.49 (7.2 – 10)	0.05 ± 0^a^	7.3 ± 0.17 (6.8 – 8.9)
2	20.13 ±0.8 (16 – 28)	54.1 ± 14.1 (40 – 68.2)	7.6 ± 0.6 (6.3 – 9.8)	0.05 ± 0^a^	7.3 ± 0.14 (6.9 – 8.8)
3	18 ± 2.12 (12 – 21)	32 ± 4 (28 – 36)	7.3 ± 0.46 (6.4 – 8.5)	0.05 ± 0^a^	7.2 ± 0.16 (6.9 – 8.9)
Swamp					
1	21.5 ± 3.6 (16 – 32)	59.8 ± 4.2 (55.7 – 64)	6.64 ± 0.7 (5.5 – 8.25)	0.045 ± 0 (0.03 – 0.05)	6.9 ± 0.06 (6.9 – 7.8)
2	31 ± 4.4 (20 – 40)	59.1 ± 1.1 (58 – 60.2)	6.67 ± 0.4 (5.8 – 7.75)	0.048 ± 0 (0.04 – 0.05)	7.1 ± 0.09 (6.8 – 7.8)
3	23.75± 3.8 (18 – 35)	66.6 ± 11.4 (55.2 – 78)	5.7 ± 0.65 (5.5 – 7)	0.048 ± 0 (0.04 – 0.05)	7.05± 0.07 (6.8 – 7.5)
Puddles					
1	24 ±1.4 (20 – 26)	40.75 ± 3.75 (37 – 44.5)	7.16 ± 0.3 (6.95 – 7.9)	0.048 ± 0 (0.04 – 0.05)	6.96± 0.09 (6.5 – 8.2)
2	21.5 ± 2.2 (16 – 24)	61.25 ± 26.2 (35 – 87.42)	7.45 ± 0.6 (6.8 – 9.1)	0.05 ± 0 (0.05 – 0.06)	6.9 ± 0.03 (6.7 – 7.2)
3	22.7 ± 4.8 (16 – 32)	35.5 ± 2.5 (33 – 38)	6.87 ± 0.2 (6.7 – 7.2)	0.07 ±0.02 (0.05 – 0.1)	6.9 ± 0.03 (6.9 – 7)

**Habitat – **Site	NO_3_ (ppm)	Temp. (°C)	Turbidity (JTU)	TDS (ppm)	

Rockpools					
1	2.2 ± 0^a^	28.02 ± 0.74 (23.6 – 31.5)	30 ± 5 (25 – 35)	37.32 ± 19.7 (24.1 – 50.5)	
2	2.2 ± 0^a^	27.52 ± 0.93 (23.5 – 36.8)	30 ± 5 (25 – 35)	36.25 ± 14.1 (26.8 – 45.7)	
3	2.2 ± 0^a^	28.12 ± 1.05 (23.5 – 37.3)	30 ± 5 (25 – 35)	21.44 ± 4 (18.8 – 24.1)	
Swamp					
1	2.2 ± 0^a^	25.48 ± 0.69 (23 – 30.3)	265 ± 65 (200 – 330)	40.07 ± 4.2 (37.3 – 42.9)	
2	2.75 ± 0.6 (2.2 – 4.4)	27.19 ± 0.82 (20 – 33.5)	265 ± 65 (200 – 330)	39.6 ± 1.1 (36.9 – 40.3)	
3	3.85±1.7 (2.2 – 8.8)	28.35 ± 1.05 (23 – 33.5)	265 ± 65 (200 – 330)	44.62 ± 11.4 (38.9 – 52.3)	
Puddles					
1	2.75±0.6 (2.2 – 4.4)	27.4 ± 0.81 (21.2 – 33.9)	105 ± 5 (100 – 110)	27.3 ± 3.75 (24.8 – 29.8)	
2	2.2 ± 0.9 (0 – 4.4)	29.56 ± 1.96 (22.1 – 60.1)	20 ± 5 (15 – 25)	41.04 ± 26.2 (23.45 – 58.6)	
3	2.2 ± 0^a^	29.75 ± 1.5 (23.5 – 36.8)	330 ± 30 (300 – 360)	23.8 ± 2.5 (22.1 – 25.5)	

^a ^Values were the same in all measurements

### The association between species and larval breeding habitats, or sampling sites within a habitat

Overall, 1,300 of the 4,174 *An. gambiae *s.l. immatures collected were randomly selected from each sampling site in each sampling day within a habitat and processed for species identification by PCR. Of these, 960 (74 %) samples were identified (351 in rock pools, 297 in the swamp, and 312 in puddles); 340 (26 %) specimens could not be diagnosed because of poor DNA samples. The 920 immatures identified were further processed as *An. gambiae *s.s. for M and S forms.

**Figure 2:**
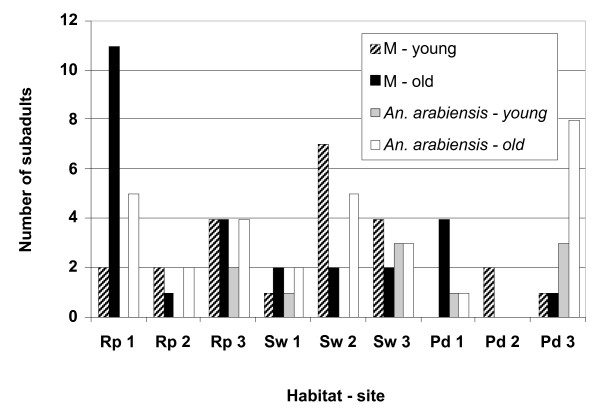
Distribution of the M forms and *An. arabiensis *(young and old immatures) sampled from three sampling sites in rock pools (Rp), swamp (Sw), and puddles (Pd) in Banambani village, Mali.

**Figure 3:**
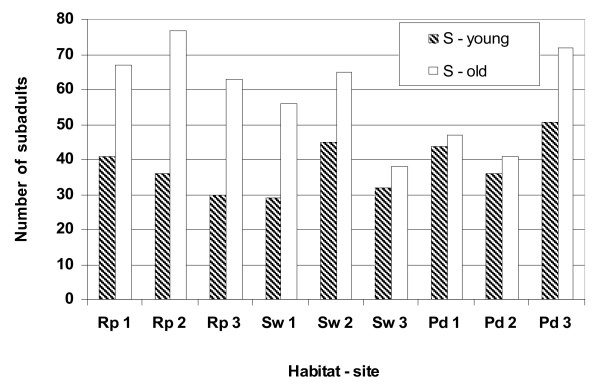
Distribution of the S forms (young and old immatures) sampled from three sampling sites in rock pools (Rp), swamp (Sw), and puddles (Pd) in Banambani village, Mali.

Immatures of *An. gambiae *s.s. were more numerous than *An. arabiensis *among all sampling sites in rock pools, the swamp and puddles where they were sympatric (Figures 2, 3). The strength of association between the species and the larval breeding habitats or sampling sites within a habitat was examined by using a type 3 analysis of the GENMOD model. This model incorporates a covariance-variance structure and calculates the association between the proportional frequencies of either *An. gambiae *s.s. and *An. arabiensis *(young and old or total immatures) among larval breeding habitats, and sampling sites within a habitat in each day's sampling and during a category of rainfall. Significant association was found between the proportional frequencies of young larvae of *An. gambiae *s.s. and *An. arabiensis *among sampling sites within each habitat (*P *< 0.05) but not among larval breeding habitats (*P *> 0.05)(Table 2). Old and total immatures of either species were similar in their proportional frequencies (*P *> 0.05) among rock pools, swamp, and puddles. Rainfall and day's sampling did not affect the relative frequencies of these immatures (*P *> 0.05).

**Table 2:** Main effects of larval breeding habitat, sampling site within each habitat, rainfall, and day on the proportional frequencies of the immatures of *An. gambiae *s.s. and *An. arabiensis *in Banambani

Source	*df*	Young		Old		Total	
		*χ*^2^	*P*	*χ*^2^	*P*	*χ*^2^	*P*
Habitat	2	0.22	0.89	0.15	0.93	0.66	0.72
Site within a habitat	2	7.69	0.02*	2.41	0.29	5.53	0.06
Category of rainfall	2	0.03	0.98	1.07	0.59	2.19	0.34
Day	1	1.37	0.24	0.33	0.57	0.01	0.92

* *P *< 0.05

### The association of the M vs. S forms and larval breeding habitats, or sampling sites within a habitat

S form immatures were more abundant than the M forms among habitats during the rainy season (Figures 2, 3). Both the M and S forms were most abundant in rock pools. The M forms were least abundant in puddles; the S forms, in the swamp. Analyzing the data as above, significant association was found between the proportional frequencies of young larvae of the M and S forms among sampling sites within a habitat (*P *< 0.05) but not among larval breeding habitats (*P *> 0.05)(Table 3). The proportional frequencies of the total immatures of the M and S forms among habitats had a borderline significance (*P *= 0.05), indicating that they were similar there. The proportional frequencies of young larvae of the M and S forms varied daily (*P *< 0.05), perhaps due to intermittent recruitment combined with high mortality and dispersal within habitats; old immatures were relatively constant (*P *> 0.05). Overall, rainfall did not affect (*P *> 0.05) the proportional frequencies of the immatures of the M and S forms.

**Table 3:** Main effects of larval breeding habitat, sampling site within a habitat, rainfall and day on the proportional frequencies of the immatures of the M and S forms in Banambani

Source	*df*	Young		Old		Total	
		*χ*^2^	*P*	*χ*^2^	*P*	*χ*^2^	*P*
Habitat	2	5.49	0.06	1.96	0.38	5.99	0.05
Site within a habitat	2	7.00	0.03*	5.57	0.06	1.91	0.38
Category of rainfall	2	4.29	0.12	2.57	0.28	4.66	0.09
Day	1	4.83	0.03*	0.34	0.56	3.84	0.05

* *P *< 0.05

### Water quality and the proportional frequencies of species

The strength of association between each of the nine physicochemical properties of water and the proportional frequencies of *An. gambiae *s.s. and *An. arabiensis *among the larval breeding habitats or sampling sites within each habitat during a category of rainfall was examined separately by using the same model as described above (Table 4). Significant *χ*^2 ^value (*P *< 0.05) was observed between the young larvae of *An. gambiae *s.s. and *An. arabiensis *and sampling sites within habitats but not among larval breeding habitats when conductivity, orthophosphate, pH and TDS had no significant effects (*P *> 0.05). The strength of association between each of these four physicochemical properties of water and proportional frequencies of species among sampling sites within each habitat was further examined by using a modified GENMOD model by eliminating the habitat effect (Table 6). Consequently, no significant effects of conductivity, orthophosphate, pH and TDS (*P *> 0.05) were observed when young larvae of *An. gambiae *s.s. and *An. arabiensis *(*P *< 0.05) were significantly associated among sampling sites within each of the larval breeding habitats. This suggested that chance or seemingly other external factors, unique in each site, influenced their association.

**Table 4:** Associations between the proportional frequencies of the immatures of *An. gambiae *s.s. and *An. arabiensis *among sampling sites within a habitat and among larval breeding habitats as influenced by each physicochemical property of water during a category of rainfall in Banambani

Source	*df*	Young		Old		Total	
		*χ*^2^	*P*	*χ*^2^	*P*	*χ*^2^	*P*

Alkalinity	1	-	-	0.70	0.404	0.45	0.503
Habitat	2			0.46	0.796	0.27	0.873
Site within a habitat	2			3.08	0.215	5.54	0.063
Category of rainfall	2			1.28	0.527	2.35	0.309
							
Conductivity	1	1.72	0.189	0.11	0.743	0.03	0.872
Habitat	2	2.68	0.262	0.01	0.993	0.28	0.869
Site within a habitat	2	7.63	0.022*	2.94	0.231	6.05	0.049*
Category of rainfall	2	1.70	0.427	1.32	0.518	2.59	0.273
							
Dissolved oxygen	1	-	-	1.00	0.317	1.75	0.186
Habitat	2			0.87	0.648	4.08	0.129
Site within a habitat	2			3.46	0.177	5.83	0.054
Category of rainfall	2			1.49	0.474	2.44	0.295
							
Nitrate	1	-	-	0.27	0.602	0.91	0.339
Habitat	2			0.11	0.946	1.28	0.526
Site within a habitat	2			2.20	0.333	4.91	0.086
Category of rainfall	2			1.25	0.536	1.89	0.389
							
Orthophosphate	1	1.33	0.249	2.34	0.126	2.00	0.157
Habitat	2	3.21	0.201	1.58	0.454	4.80	0.091
Site within a habitat	2	8.24	0.016*	0.88	0.645	4.38	0.112
Category of rainfall	2	0.68	0.713	1.38	0.501	2.48	0.289
							
pH	1	0.34	0.559	2.38	0.123	3.20	0.074
Habitat	2	1.08	0.583	0.30	0.861	1.34	0.511
Site within a habitat	2	7.71	0.021*	1.80	0.406	4.81	0.090
Category of rainfall	2	0.54	0.762	1.22	0.543	2.86	0.239
							
Temperature	1	-	-	1.14	0.287	3.47	0.063
Habitat	2			0.40	0.817	2.65	0.265
Site within a habitat	2			1.58	0.454	5.90	0.052
Category of rainfall	2			1.44	0.486	4.38	0.112
							
TDS	1	2.00	0.157	0.24	0.626	0.02	0.895
Habitat	2	2.93	0.231	0.05	0.977	0.33	0.849
Site within a habitat	2	7.61	0.022*	3.17	0.205	5.77	0.056
Category of rainfall	2	1.98	0.371	1.31	0.521	2.63	0.268
							
Turbidity	1	-	-	2.27	0.132	2.57	0.109
Habitat	2			2.10	0.350	1.71	0.425
Site within a habitat	2			0.26	0.878	2.91	0.234
Category of rainfall	2			1.61	0.448	2.65	0.266

* *P *< 0.05, "-" not applicable

**Table 5:** Associations between the proportional frequencies of the immatures of the M and S forms among sampling sites within a habitat, or among larval breeding habitats as influenced by each physicochemical property of water during a category of rainfall in Banambani

Source	*df*	Young		Old		Total	
		*χ*^2^	*P*	*χ*^2^	*P*	*χ*^2^	*P*

Alkalinity	1	3.34	0.068	0.22	0.636	2.64	0.104
Habitat	2	4.59	0.101	2.39	0.303	4.39	0.111
Site within a habitat	2	6.52	0.038*	5.61	0.060	3.50	0.174
Category of rainfall	2	4.38	0.112	2.73	0.255	4.43	0.109
							
Conductivity	1	1.59	0.208	0.60	0.438	0.06	0.812
Habitat	2	3.20	0.202	2.82	0.244	3.55	0.169
Site within a habitat	2	6.51	0.039*	5.29	0.071	1.35	0.508
Category of rainfall	2	3.01	0.222	2.47	0.291	3.79	0.812
							
Dissolved oxygen	1	0.59	0.443	0.03	0.872	0.85	0.357
Habitat	2	5.02	0.081	2.14	0.343	5.28	0.072
Site within a habitat	2	6.31	0.043*	5.69	0.058	1.79	0.409
Category of rainfall	2	3.45	0.179	2.54	0.280	3.97	0.137
							
Nitrate	1	1.05	0.305	0.56	0.455	0.01	0.906
Habitat	2	5.48	0.065	2.44	0.295	5.02	0.081
Site within a habitat	2	5.77	0.056	5.28	0.071	1.22	0.543
Category of rainfall	2	5.28	0.071	2.23	0.327	3.77	0.152
							
Orthophosphate	1	-	-	0.26	0.610	-	-
Habitat	2			2.32	0.314		
Site within a habitat	2			5.05	0.080		
Category of rainfall	2			2.58	0.275		
							
pH	1	0.73	0.393	1.14	0.285	1.68	0.195
Habitat	2	4.27	0.118	1.31	0.521	4.08	0.130
Site within a habitat	2	5.71	0.058	6.23	0.044*	0.88	0.645
Category of rainfall	2	5.28	0.203	2.21	0.332	3.14	0.209
							
Temperature	1	0.01	0.934	1.64	0.201	1.78	0.182
Habitat	2	4.75	0.093	2.85	0.241	5.54	0.063
Site within a habitat	2	6.39	0.041*	5.69	0.058	1.37	0.505
Category of rainfall	2	3.45	0.179	2.91	0.234	2.81	0.245
							
TDS	1	1.85	0.174	0.66	0.416	0.01	0.927
Habitat	2	3.18	0.204	2.88	0.237	4.01	0.135
Site within a habitat	2	6.65	0.036*	5.27	0.072	1.28	0.526
Category of rainfall	2	2.89	0.236	2.47	0.290	3.75	0.153
							
Turbidity	1	0.04	0.846	2.27	0.132	0.08	0.781
Habitat	2	4.79	0.091	2.10	0.350	5.12	0.077
Site within a habitat	2	5.76	0.056	0.26	0.878	1.07	0.586
Category of rainfall	2	3.58	0.167	1.61	0.448	2.90	0.235

* *P *< 0.05, "-" Not applicable

**Table 6:** Associations between the proportional frequencies of the immatures of *An. gambiae *s.s. and *An. arabiensis *among sampling sites within a habitat as influenced by each physicochemical property of water during a category of rainfall in Banambani

Source	*df*	Young		Old	
		*χ*^2^	*P*	*χ*^2^	*P*

Conductivity	1	0.01	0.906	0.15	0.702
Site within a habitat	2	7.12	0.028*	3.00	0.224
Category of rainfall	2	0.36	0.835	1.37	0.504
					
Orthophosphate	1	0.77	0.381	1.88	0.170
Site within a habitat	2	7.27	0.026*	0.56	0.756
Category of rainfall	2	0.01	0.995	1.54	0.464
					
pH	1	0.30	0.585	2.22	0.163
Site within a habitat	2	6.95	0.031*	1.82	0.403
Category of rainfall	2	0.29	0.867	1.52	0.136
					
TDS	1	0.06	0.806	3.23	0.601
Site within a habitat	2	7.08	0.029*	3.23	0.199
Category of rainfall	2	0.35	0.841	1.36	0.506

* *P *< 0.05

The old immatures of *An. gambiae *s.s. and *An. arabiensis *appeared very tolerant to the temporal changes of all physicochemical variables measured (*P *> 0.05) (Table 4). Significant association between the proportional frequencies of the total *An. gambiae *s.s. and *An. arabiensis *among sampling sites within habitats (*P *= 0.049) was found when conductivity had no significant effect (*P *> 0.05). Further analysis (Table 6) revealed that conductivity had no significant effect on the proportional frequencies of these sibling species among sampling sites within habitats, and that chance or seemingly other external factors, unique in each site, influenced their relative frequencies. Overall, rainfall did not have any effect (*P *> 0.05).

### Water quality and the proportional frequencies of the M and S forms

Analyzing the data as above (Table 5), significant association between the proportional frequencies of young larvae of the M and S forms of *An. gambiae *s.s. among sampling sites within each habitat (*P *< 0.05) but not among larval breeding habitats (*P *> 0.05) was observed when alkalinity, conductivity, D.O., temperature, and TDS had no significant effects. The strength of association between each of these five physicochemical properties of water and proportional frequencies of molecular forms among sampling sites within each habitat was further examined by eliminating the habitat effect in the GENMOD model as described above (Table 7). Interestingly, conductivity and TDS had significant effects (*P *< 0.05) when the proportional frequencies of young larvae of the M and S forms among sampling sites within habitats were significantly associated (*P *< 0.05).

**Table 7:** Associations between the proportional frequencies of the young and old immatures of the M and S forms among sampling sites within a habitat as influenced by each physicochemical property of water during a category of rainfall in Banambani

Source	*df*	Young		Old	
		*χ*^2^	*P*	*χ*^2^	*P*

Alkalinity	1	3.64	0.056	0.00	0.958
Site within a habitat	2	3.43	0.179	5.16	0.235
Category of rainfall	2	3.43	0.180	2.90	0.958
					
Conductivity	1	5.38	0.020*	0.27	0.603
Site within a habitat	2	6.29	0.043*	4.50	0.105
Category of rainfall	2	2.69	0.259	2.92	0.232
					
D.O.	1	0.02	0.896	0.74	0.391
Site within a habitat	2	4.58	0.101	5.27	0.072
Category of rainfall	2	2.25	0.325	3.19	0.203
					
pH	1	1.23	0.268	1.17	0.279
Site within a habitat	2	4.28	0.118	5.64	0.059
Category of rainfall	2	1.88	0.392	2.28	0.319
					
Temperature	1	0.01	0.940	1.22	0.269
Site within a habitat	2	4.27	0.118	5.25	0.072
Category of rainfall	2	2.16	0.339	3.27	0.195
					
TDS	1	5.70	0.017*	0.31	0.580
Site within a habitat	2	6.50	0.039*	4.47	0.107
Category of rainfall	2	2.67	0.263	2.93	0.231

* *P *< 0.05

Older larvae and pupae of the M and S forms appeared more resilient to most of the water properties (*P *> 0.05) except for pH. A significant association was observed between the proportional frequencies of old immatures of the M and S forms among sampling sites within habitats (*P *< 0.04) when pH had no significant effect (*P *> 0.05) (Tables 5, 7).

## Discussion

A combination of factors may have contributed to the relative frequency patterns of the immatures of *An. gambiae *s.s., its M and S forms, and *An. arabiensis *observed at Banambani village, Mali: 1) water quality within habitats in the presence of seemingly other external factors, 2) oviposition patterns, and 3) differential survivorship among species and forms.

*Water quality and other factors*. Conductivity and TDS have significant effects when the proportional frequencies of young larvae of the M and S forms among sampling sites within habitats in Banambani are significantly associated (Table 7). Conductivity is a measure of the inorganic materials and ions in water, whereas TDS are the sum of all dissolved (organic or inorganic) and suspended solids in water [33]. As TDS increase, conductivity also increases. Pollution from irrigation, along with materials related to flooding after heavy rains, influence conductivity and TDS, and may have restricted larval population to sites within habitats that have tolerable ecological regimens. Reisen *et al*. [34] find that high conductivity may have altered the age structure of *Culex tarsalis *in Kern County, California.

Alkalinity, D.O., orthophosphate, pH, nitrate, temperature and turbidity have no effects on niche partitioning of the immatures of *An. gambiae *s.s., its M and S forms, and *An. arabiensis *because these physicochemical properties, except turbidity, are mainly similar among sampling sites within habitats (Table 1). Sagnon *et al*. [35] do not also detect strong associations between the species or molecular forms and any physicochemical variables of water, except conductivity, in Burkina Faso. Despite the insignificant effects of these physicochemical properties of water on mosquito immatures, significant association is found between the proportional frequencies of young larvae of *An. arabiensis, An. gambiae *s.s. and its M and S forms among sampling sites within habitats but not among larval breeding habitats. Thus, water properties influence growth rates of larvae, although other unmeasured factors may also be important.

It is speculated that the M and S forms contrast significantly in their way of exploiting limiting resources in their habitats [5,18]. This is not shown in the current study and is consistent with Diabate *et al*. [20], although it is possible that the forms differ in utilizing limiting resources along with other variables that are not measured. Candidate correlates include bacterial composition [16], chlorophyll [36], detritus [37], and maize pollen (*Zea mays*)[38,39] which are also sources of food for the larvae. Ye-Ebiyo *et al*. [38] observe that *An. arabiensis *larvae readily ingest wind-borne maize pollens that are nutritious enough to support their development. In Banambani, there are several maize plants near rock pools, thus pollens can be dispersed by the wind within the village because every maize plant can produce 14–50 million grains [40] and around 500 thousand pollen grains may remain airborne ≥ 60 m from maize plants that produce ~25 million grains [41]. These are interesting topics to be investigated for members of *An. gambiae *s.l. in Mali.

*Oviposition patterns*. Significant association between the young larvae of *An. gambiae *s.s., its M and S forms, and *An. arabiensis *and sampling sites in each of the larval breeding habitats supports the hypothesis that oviposition patterns and behavior of *An. gambiae *s.l. may have influenced microhabitat colonization [5,9]. It also suggests differential selection of oviposition sites within habitats by adult females of *An. gambiae *s.s. and *An. arabiensis*. Significant daily variation in the relative frequencies of young larvae of the M and S forms seems to reflect changes in the oviposition behavior of *An. gambiae *s.s. Alternatively, the microbial fauna in larval breeding habitats of *An. gambiae *s.l. probably release volatiles that may be used as oviposition cues or deterrents [14]. *Aedes *and *Culex *mosquitoes exhibit a differential response to oviposition media based on the composition of microbial species [42-44]. This is an interesting area for future investigation for members of *An. gambiae *s.l.

Coluzzi [45] and Powell *et al*. [46] have emphasized the importance of historical changes in human-mosquito associations. It appears difficult to determine the association of species and forms based on distance to houses where adult females have their blood meal host or resting shelter in the village, because there are no differences in their frequencies among habitats. Previous mark-release-recapture (MRR) experiments revealed insignificant association between species and forms of female adult mosquitoes and larval abundance in the habitats adjoining them in 1997 and 1998 mid-rainy seasons in Banambani [17]. This was consistent with the MRR study in the village in 1993–1994 [23]. Costantini *et al*. [47] reported occasional intervillage exchange of adult *An. gambiae *s.l. after heavy rains in Ouagadougou, Burkina Faso; Touré *et al*. [24] surmised that the same would be true of Banambani, and in fact some were observed by Taylor *et al*. [48].

*Differential survivorship among species and forms*. Daily variation on the proportional frequency of young larvae of the M and S forms implies differential survivorship among life stages of immatures among larval breeding habitats in Banambani. Reisen *et al*. [49] also observe unequal effects of mortality factors among life stages of *Anopheles culicifacies *that contribute to their disproportionate distribution in Pakistan. Mortality of *An. gambiae *s.l. immatures in Kenya occurs mainly among third and fourth instars in marshes, and pupal mortality is relatively high in temporary pools [22]. Although, survivorship estimates of *An. gambiae *s.l. are found similar among the larval breeding habitats in Banambani [23], emergence rate of the S form is higher than that of the M form in puddles and quarries [20].

## Conclusion

Conductivity and TDS have significant effects when the proportional frequencies of young larvae of the M and S forms among sampling sites within each type of larval breeding habitats (rock pools, the swamp and puddles) in Banambani, Mali are significantly associated. Alkalinity, D.O., orthophosphate, pH, nitrate, temperature, and turbidity have no differential effects on niche partitioning of the immatures of *An. gambiae *s.s., its M and S forms, and *An. arabiensis *because these physicochemical properties, except turbidity, are mainly similar among sampling sites within and among larval breeding habitats. It may be that the distribution of these mosquito immatures reflects adaptive differences of adult females, their oviposition patterns, differential survivorship of immatures, and the influence of water quality among sampling sites within habitats in the presence of some other environmental factors. These include bacterial composition, chlorophyll, detritus, and maize pollens that need to be further investigated.

## Authors' contributions

FE conceived the design of the study, carried out the field and molecular work, performed all data analyses, and wrote the manuscript. FT initially helped the PCR-based diagnostic in identifying the M and S forms, and critiqued the manuscript. YT critiqued the manuscript and together with GD facilitated in the coordination of field work and DNA extractions that were done in Mali. GL provided the laboratory facilities and reagents for all molecular work. CT participated in the design of the study, coordination of collaborators, and helped to revise the manuscript. All authors read and approved the final manuscript.
